# Safety and Effectiveness in 400 Image-Guided Spleen Biopsies from the DeGIR Registry

**DOI:** 10.1007/s00270-026-04517-0

**Published:** 2026-06-29

**Authors:** R. Ocker-Serger, M. Opitz, L. Klüner, M. Drews, D. Rosok, L. Salhoefer, M. Forsting, J. Haubold, B. M. Schaarschmidt, S. Zensen

**Affiliations:** Institute of Diagnostic and Interventional Radiology and Neuroradiology, University Hospital Essen, Essen, Germany

**Keywords:** Splenic biopsy, Image-guided biopsy, Complication rate, Diagnostic yield

## Abstract

**Purpose:**

To evaluate technical success, diagnostic yield, and complication rates of image-guided percutaneous splenic biopsies based on multicenter registry data from the German Society for Interventional Radiology and Minimally Invasive Therapy.

**Materials and Methods:**

This retrospective multicenter study analyzed image-guided percutaneous splenic biopsies documented in the prospective DeGIR registry between 2018 and 2024. Technical success was defined as needle placement within the target lesion. Diagnostic yield was defined as histologically adequate samples enabling clinical diagnosis.

**Results:**

Four hundred splenic biopsies from 92 centers were included. Technical success was achieved in 99.00% of procedures. The complication rate was 5.00%, including 2.50% major complications with no procedure-related deaths. Complication rates were higher for procedures performed under local anesthesia than analgesic sedation (*p* = 0.042).

Histopathological reports were available for 338 procedures, resulting in a diagnostic yield of 90.83%. Diagnostic yield increased with the number of biopsy samples, reaching a maximum with three samples. Procedures performed under analgesic sedation showed a higher diagnostic yield than those under local anesthesia (*p* = 0.019).

**Conclusion:**

Image-guided percutaneous splenic biopsy is a safe and effective diagnostic procedure with high technical success and diagnostic yield. Analgesic sedation and obtaining up to three biopsy samples may improve diagnostic performance without increasing complication rates.

**Graphical Abstract:**

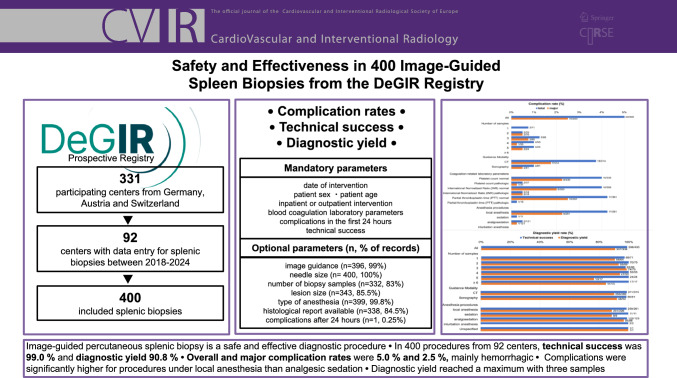

## Introduction

Histopathological evaluation is essential for differentiating inflammatory, infectious, and neoplastic splenic processes, particularly in patients with hematologic or oncologic disease, where imaging may lack sufficient specificity and histological confirmation becomes decisive for therapy [1, 2]. Although diagnostic splenectomy is still performed in selected cases [3, 4], it is generally considered as a last-resort procedure. Given the considerable perioperative risks and the increased lifelong susceptibility to infection following splenectomy [5, 6], image-guided splenic biopsy is favored as a minimally invasive alternative whenever feasible.

Image-guided spleen punctures, typically performed under ultrasound (US) or computed tomography (CT) guidance, are well-established diagnostic procedures for evaluating focal or diffuse splenic lesions when noninvasive imaging remains inconclusive. Both modalities are used for fine-needle aspiration and core needle biopsy, with ultrasound guidance being more common due to real-time visualization and lack of radiation, while CT guidance is preferred for deep or poorly visualized lesions [7, 8].

Although image-guided spleen punctures are established and generally safe diagnostic procedures [9, 10], they are still approached with caution due to the spleen’s rich vascularization and perceived risk of hemorrhage.

The German Society for Interventional Radiology and Minimally Invasive Therapy (DeGIR), part of the Cardiovascular and Interventional Radiological Society of Europe (CIRSE), operates a prospective multicenter registry designed for quality assurance and research, currently encompassing a broad network of centers across Germany, Austria, and Switzerland. This registry provides a comprehensive foundation for evaluating real-world clinical practice in interventional radiology [11–13]. The objective of this study was to assess technical success, diagnostic yield, and complication rates of image-guided percutaneous spleen punctures performed across DeGIR registry sites between 2018 and 2024.

## Material and Methods

### Data Source and Study Design

This multicenter retrospective analysis evaluated image-guided percutaneous spleen biopsies documented in the DeGIR registry from January 2018 to December 2024. The registry is prospectively maintained with centralized ethical approval and additional local approvals as applicable. Participating institutions submitted standardized routine data via a web-based platform (samedi GmbH, Berlin, Germany).

### Inclusion Criteria and Definitions

**Fig. 1:**
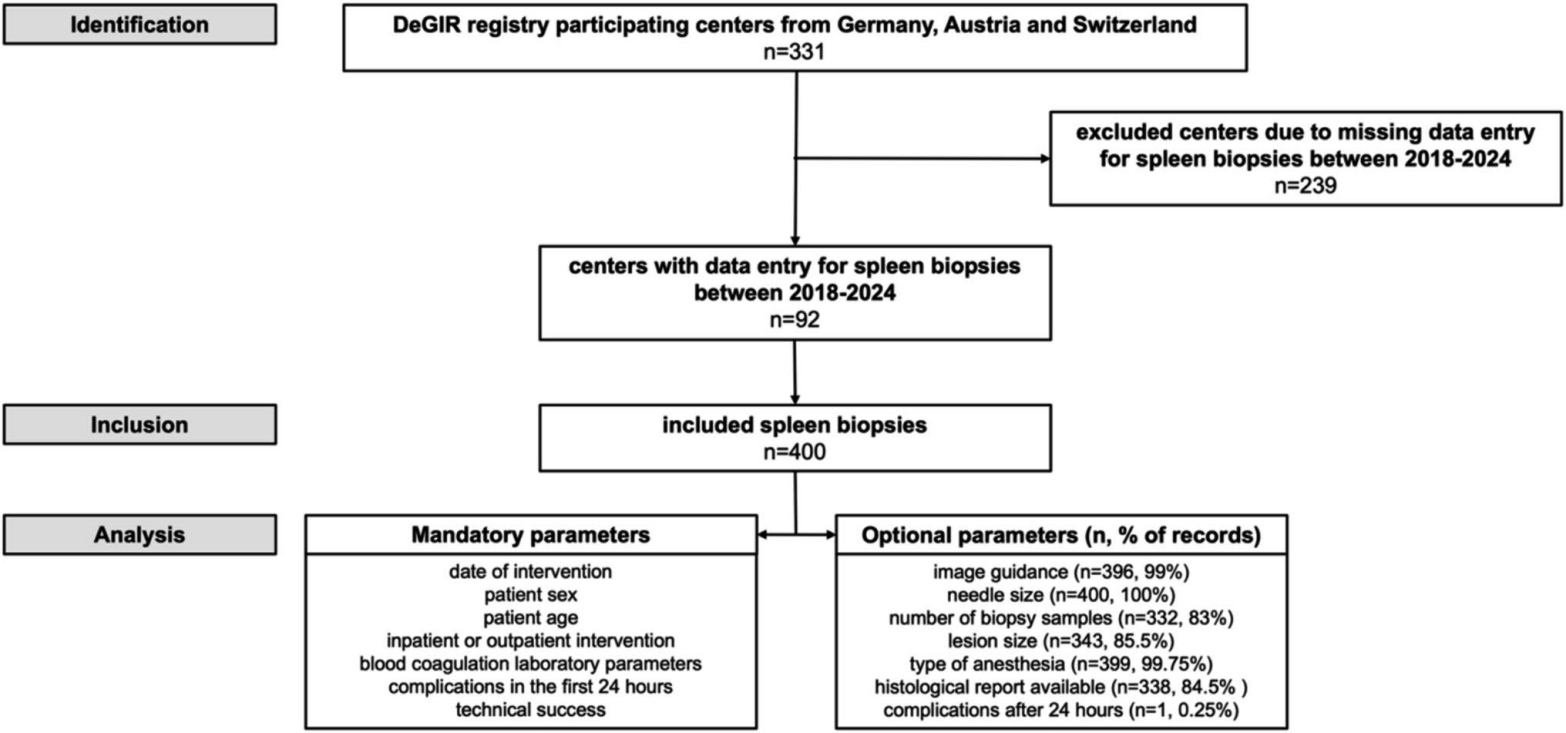
Study flow chart

All consecutive image-guided percutaneous splenic biopsies documented in the DeGIR registry during the study period were included without predefined clinical inclusion or exclusion criteria, reflecting real-world practice across participating centers. The registry dataset included a combination of mandatory and optional variables, which are summarized in Fig. 1. Technical success was defined as visually confirmed biopsy needle placement within the target lesion. The coagulation parameters (platelet count, INR, PTT) were recorded as categorical variables either pathological or normal, based on the local laboratory standards at each participating center. No predefined numerical thresholds were specified in the registry.

Complications were classified according to the Society of Interventional Radiology (SIR) severity grading system, distinguishing between minor (grades A–B) and major (grades C–F) events [14], and categorized as either early (within 24 h) or delayed (onset after 24 h). The updated CIRSE classification system [15] is currently regarded as the standard framework for grading complications in interventional radiology.

Diagnostic yield was considered achieved when a procedure provided histologically adequate material enabling a definitive clinical diagnosis. As histological reports were documented only when entered as an optional registry field, diagnostic yield was calculated from the subset of cases with available reports (*n* = 338). Lesion texture was categorized at the time of biopsy based on imaging appearance. Biopsy technique was recorded in the registry and categorized as core needle biopsy and fine-needle aspiration. Core needle biopsy included Tru-Cut and vacuum-assisted biopsy techniques. The registry did not include information on the use of coaxial systems. Cases with missing data were excluded from the respective analyses. All analyses were conducted using available data on a per-variable basis. No imputation of missing data was performed.

### Statistical Analysis

Statistical analysis was performed using SPSS software, version 26.0 (IBM Corp., Armonk, NY, USA). The distribution of continuous variables was evaluated using the Kolmogorov–Smirnov test. Variables not normally distributed are presented as median with interquartile range (IQR). Categorical variables, such as technical success, diagnostic yield, and complication rates, were compared using the chi-square test, while non-normally distributed continuous variables were analyzed with the Mann–Whitney U test. A p value < 0.05 was considered statistically significant. All subgroup analyses were exploratory; therefore, no formal adjustment for multiple testing was applied, and reported p values should be interpreted descriptively. Multivariable logistic regression was not performed due to the low number of events and incomplete data across several variables. Associations were, therefore, assessed using descriptive statistics and univariable analyses.

## Results

### Patient Characteristics

Between 2018 and 2024, a total of 400 image-guided percutaneous spleen biopsies were reported to the DeGIR registry by 92 centers (Fig. 1). The median number of procedures per center was 2 (IQR: 1–4). The median patient age was 63 years (IQR: 51–74). Information on interdisciplinary tumor board decisions was available for 34.25% of procedures (137/400), with biopsy recommended in 96.35% (132/127) of cases. Most procedures were performed in inpatient setting (94.25%) (Table 1).

**Table 1:** Frequency of percutaneous image-guided spleen biopsies from 2018 to 2024 by setting and gender distribution

	2018	2019	2020	2021	2022	2023	2024	All
All	43	46	42	39	50	47	133	400
Setting								
Inpatient	41	42	42	36	47	43	126	377
	95.35%	91.30%	100.00%	92.31%	94.00%	91.49%	94.74%	94.25%
Outpatient	2	4	0	3	3	4	7	23
	4.65%	8.70%	0.00%	7.69%	6.00%	8.51%	5.26%	5.75%
Gender distribution								
Female	13	25	17	17	28	20	69	189
	30.23%	54.35%	40.48%	43.59%	56.00%	42.55%	51.88%	47.25%
Male	30	21	25	22	22	27	64	211
	69.77%	45.65%	59.52%	56.41%	44.00%	57.45%	48.12%	52.75%

### Lesion and Biopsy Characteristics

CT was the predominant image guidance modality (78.50%), followed by US (20.25%). The median lesion size was 30 mm (IQR: 15–49 mm). The majority of procedures were performed using core needle biopsy techniques (85.75%; *n* = 343), while fine-needle aspiration was used in 13.25% of cases (*n* = 53). Most lesions were solid (65.50%, 262/400). The number of biopsy samples was documented only for core needles biopsies with a median number of 3 samples (IQR: 2–4).

### Complications

**Fig. 2:**
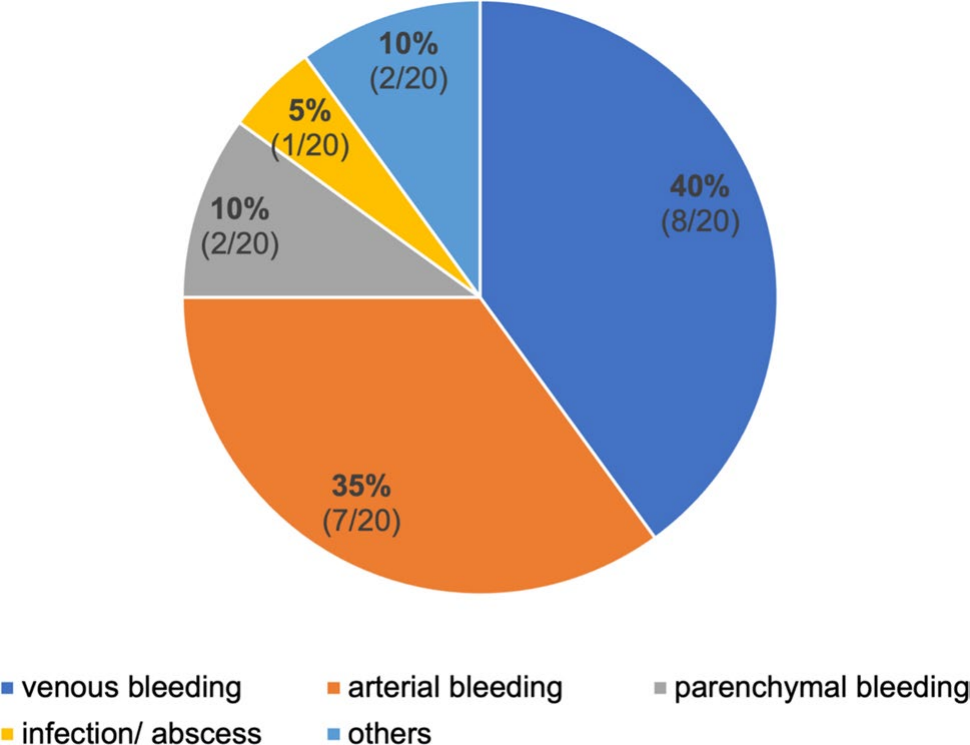
Complications of percutaneous image-guided spleen biopsies differentiated by complication type

The overall complication rate within 24 h post-procedure was 5.00% (20/400). In total, 2.50% (10/400) of complications were minor, including four cases classified as SIR grade A (no therapy, no sequelae), and six cases as grade B (requiring symptomatic therapy or observation). Major complications occurred in 2.50%, including five cases grade C and five cases grade D complications. There was no permanent health damage (Grade E) or death (Grade F) within 24 h. The most frequent complication type was bleeding (Fig. 2). The cause of bleeding (venous, parenchymal, arterial) was documented in the register by the attending physician.

In one case, delayed complications (> 24 h post-procedure) were reported, which were classified as major (Grade D) venous bleeding.

The overall complication rate was 5.31% in inpatient setting and 0.00% in outpatient procedures. When comparing image guidance modalities, CT-guided biopsies were associated with comparable complication rates than US-guided procedures (3.75 vs. 1.00%; OR = 0.97, 95%CI 0.31–2.99; *p* = 0.952); major complications were also comparable (1.75 vs. 0.50%; OR = 0.901, 95%CI 0.18–4.42; *p* = 0.897). Lesion size was not significantly associated with total or major complication rates. Across all defined size categories, complication rates ranged between 0.75% and 1.75%. No significant differences in complication rates were observed for pathological platelet counts (0.50 vs. 4.00%; OR = 0.89, 95%CI 0.20–4.04; *p* = 0.882), pathological INR values (0.50 vs. 4.00%; OR = 0.38, 95%CI 0.08–1.81; *p* = 0.210), or pathological PTT values (0.25 vs. 4.25%; OR = 0.71, 95%CI 0.09–5.74; *p* = 0.749).

**Fig. 3:**
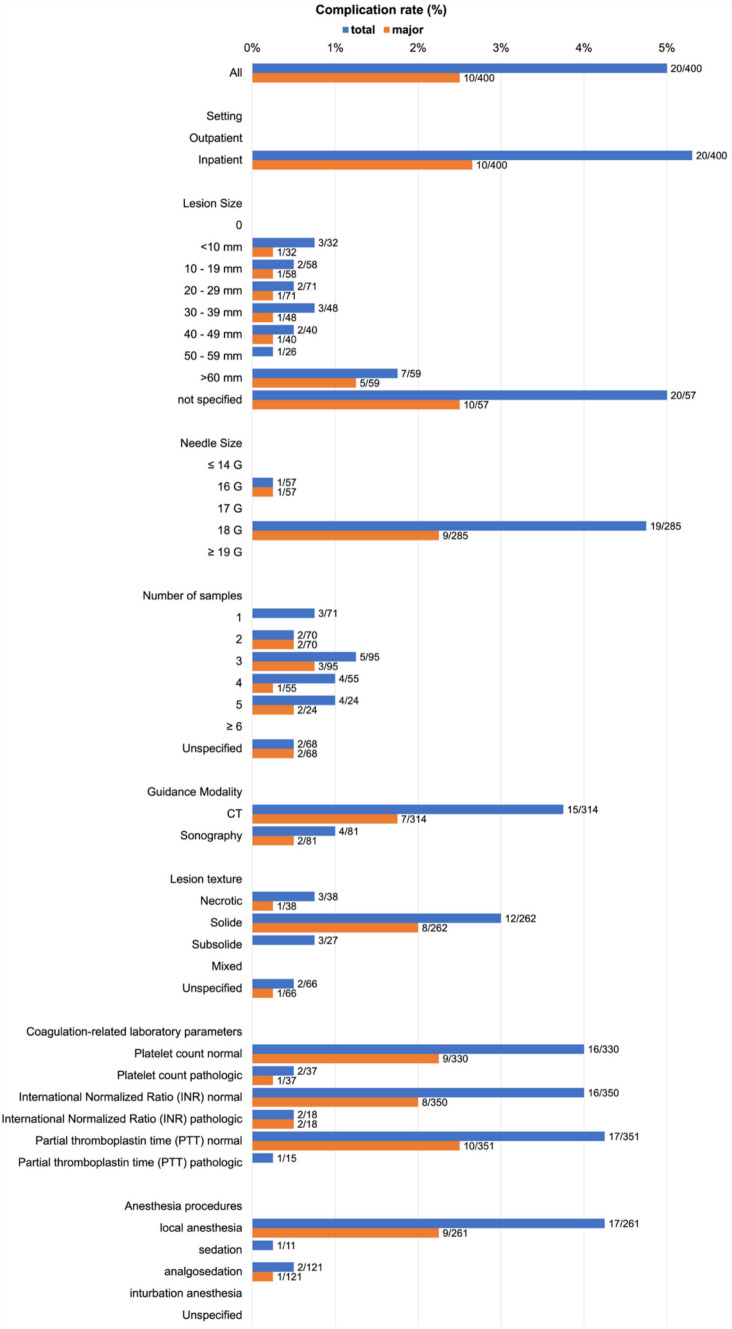
Complications of percutaneous image-guided spleen biopsies

Procedures performed under local anesthesia had significantly higher complication rates than procedures performed under analgesic sedation (4.25 vs. 0.5%; OR = 4.15, 95%CI 0.94–8.24; *p* = 0.042) (Table 2, Fig. 3).

**Table 2:** Complications within 24 h of percutaneous image-guided spleen biopsies

	Procedures	Complications			
	(n)	Total (*n*)	(%)	Major according to SIR classification [14] (*n*)	(%)
All	400	20	5.00%	10	2.50%
Setting					
Outpatient	23	0	0.00%	0	0.00%
Inpatient	377	20	5.31%	10	2.65%
Lesion Size					
0	9	0	0.00%	0	0.00%
< 10 mm	32	3	0.75%	1	0.25%
10–19 mm	58	2	0.50%	1	0.25%
20–29 mm	71	2	0.50%	1	0.25%
30–39 mm	48	3	0.75%	1	0.25%
40–49 mm	40	2	0.50%	1	0.25%
50–59 mm	26	1	0.25%	0	0.00%
≥ 60 mm	59	7	1.75%	5	1.25%
Unspecified	57	20	5.00%	10	2.50%
Needle Size					
*Core needle biopsies*					
≤ 14 G	5	0	0.00%	0	0.00%
16 G	50	0	0.00%	0	0.00%
17 G	16	0	0.00%	0	0.00%
18 G	260	19	7.31%	9	3.46%
≥ 19 G	12	0	0.00%	0	0.00%
*Fine-needle aspiration*					
≤ 14 G	6	0	0.00%	0	0.00%
16 G	6	1	16.67%	1	16.67%
17 G	5	0	0.00%	0	0.00%
18 G	23	0	0.00%	0	0.00%
≥ 19 G	13	0	0.00%	0	0.00%
Number of samples obtained (core needle biopsies)					
1	71	3	0.75%	0	0.00%
2	70	2	0.50%	2	0.50%
3	95	5	1.25%	3	0.75%
4	55	4	1.00%	1	0.25%
5	24	4	1.00%	2	0.50%
≥ 6	17	0	0.00%	0	0.00%
Unspecified	68	2	0.50%	2	0.50%
Guidance Modality					
CT	314	15	3.75%	7	1.75%
Sonography	81	4	1.00%	2	0.50%
Mixed	1	1	100.00%	1	100.00%
Unspecified	4	0	0.00%	0	0.00%
Lesion texture					
Necrotic	38	3	0.75%	1	0.25%
Solid	262	12	3.00%	8	2.00%
Subsolid	27	3	0.75%	0	0.00%
Mixed	7	0	0.00%	0	0.00%
Unspecified	66	2	0.50%	1	0.25%
Coagulation-related laboratory parameters					
Platelet count normal	330	16	4.00%	9	2.25%
Platelet count pathologic	37	2	0.50%	1	0.25%
International Normalized Ratio (INR) normal	350	16	4.00%	8	2.00%
International Normalized Ratio (INR) pathologic	18	2	0.50%	2	0.50%
Partial thromboplastin time (PTT) normal	351	17	4.25%	10	2.50%
Partial thromboplastin time (PTT) pathologic	15	1	0.25%	0	0.00%
Anesthesia procedures					
Local anesthesia	261	17	4.25%	9	2.25%
Sedation	11	1	0.25%	0	0.00%
Analgosedation	121	2	0.50%	1	0.25%
Intubation anesthesia	2	0	0.00%	0	0.00%
Unspecified	1	0	0.00%	0	0.00%

### Technical Success

**Fig. 4:**
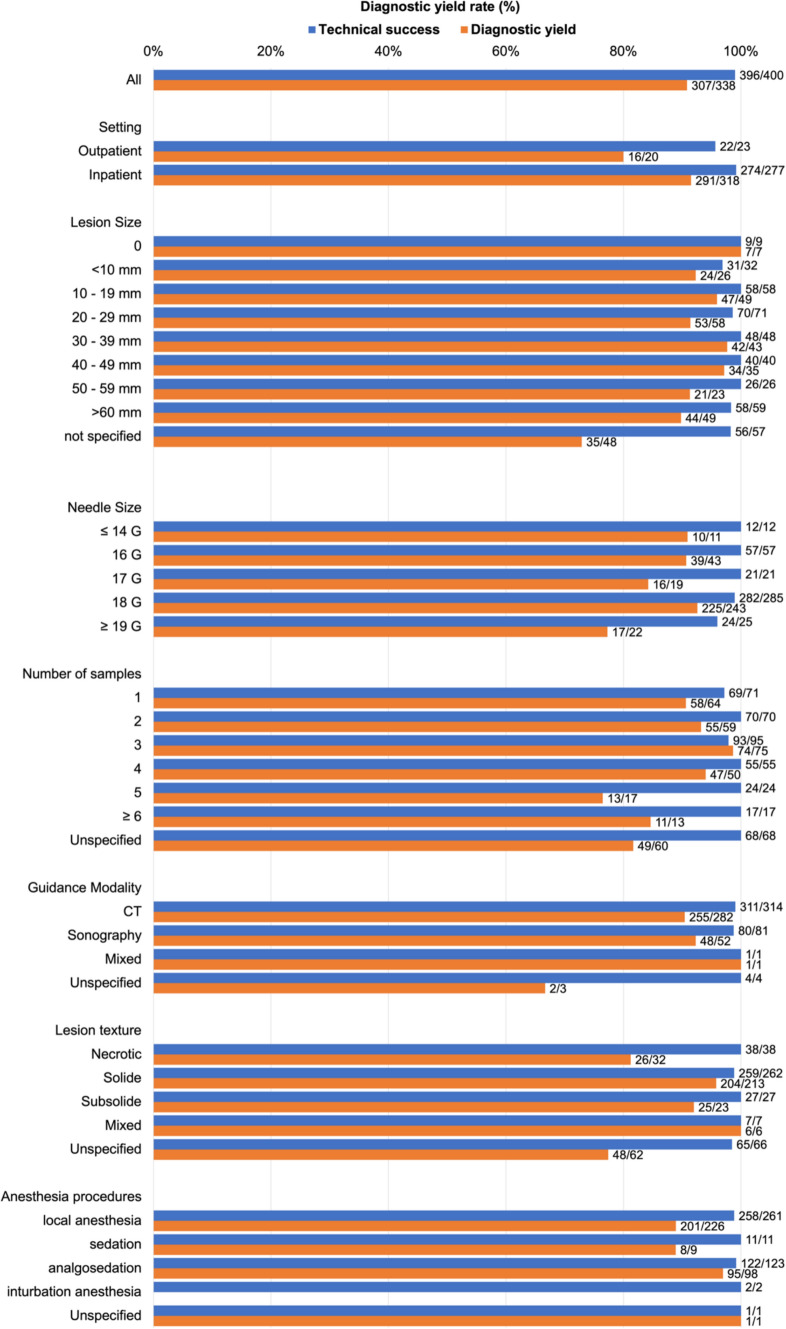
Technical success and histological representativeness rates of percutaneous image-guided spleen biopsies

The overall technical success rate was 99.00%. Technical success rates were equally high for inpatients and outpatients (OR = 0.18, 95%CI 0.02–1.77; *p* = 0.096), CT guidance and US guidance (OR = 1.30, 95%CI 0.13–2.6; *p* = 0.823), as well as solid and necrotic lesion texture (OR = 0.87, 95%CI 0.84–0.91; *p* = 0.507) (Table 3, Fig. 4).

**Table 3:** Technical success and diagnostic yield of percutaneous image-guided spleen biopsies

	Procedures	Technical success		Diagnostic yield		
	n	Procedures (*n*)	rate (%)	Report available (*n*)	Diagnosis possible (*n*)	rate (%)
All	400	396	99.00%	338	307	90.83%
Setting						
Outpatient	23	22	95.65%	20	16	80.00%
Inpatient	377	374	99.20%	318	291	91.51%
Lesion Size						
0	9	9	100.00%	7	7	100.00%
< 10 mm	32	31	96.88%	26	24	92.31%
10–19 mm	58	58	100.00%	49	47	95.92%
20–29 mm	71	70	98.59%	58	53	91.38%
30–39 mm	48	48	100.00%	43	42	97.67%
40–49 mm	40	40	100.00%	35	34	97.14%
50–59 mm	26	26	100.00%	23	21	91.30%
≥ 60 mm	59	58	98.31%	49	44	89.80%
Unspecified	57	56	98.25%	48	35	72.92%
Needle Size						
*Core needle biopsies*						
≤ 14 G	5	5	100.00%	4	3	75.00%
16 G	50	50	100.00%	37	34	91.89%
17 G	16	16	100.00%	15	12	80.00%
18 G	260	257	98.85%	219	205	93.61%
≥ 19 G	12	12	100.00%	10	8	80.00%
*Fine-needle aspiration*						
≤ 14 G	6	6	100.00%	6	6	100.00%
16 G	6	6	100.00%	5	4	80.00%
17 G	5	5	100.00%	4	4	100.00%
18 G	23	23	100.00%	22	18	81.82%
≥ 19 G	13	12	92.31%	12	9	75.00%
Number of samples obtained (core needle biopsies)						
1	71	69	97.18%	64	58	90.63%
2	70	70	100.00%	59	55	93.22%
3	95	93	97.89%	75	74	98.67%
4	55	55	100.00%	50	47	94.00%
5	24	24	100.00%	17	13	76.47%
≥ 6	17	17	100.00%	13	11	84.62%
Unspecified	68	68	100.00%	60	49	81.67%
Guidance Modality						
CT	314	311	99.04%	282	255	90.43%
Sonography	81	80	98.77%	52	48	92.31%
Mixed	1	1	100.00%	1	1	100.00%
Unspecified	4	4	100.00%	3	2	66.67%
Lesion texture						
Necrotic	38	38	100.00%	32	26	81.25%
Solid	262	259	98.85%	213	204	95.77%
Subsolid	27	27	100.00%	25	23	92.00%
Mixed	7	7	100.00%	6	6	100.00%
Unspecified	66	65	98.48%	62	48	77.42%
Anesthesia procedures						
Local anesthesia	261	258	98.85%	226	201	88.94%
Sedation	11	11	100.00%	9	8	88.89%
Analgosedation	123	122	99.19%	98	95	96.94%
Intubation anesthesia	2	2	100.00%	2	0	0.00%
Unspecified	1	1	100.00%	1	1	100.00%

### Diagnostic Yield

The overall diagnostic yield was 90.83%, with outpatients achieving similar rates to inpatients (OR = 2.69, 95% CI 0.84–8.63; *p* = 0.08). Diagnostic yield was comparable between CT- and US-guided procedures (OR = 0.79, 95% CI 0.26–2.35; *p* = 0.667). Subsolid lesions showed a slightly, but not significantly, lower diagnostic yield compared to solid lesions (OR = 1.97, 95% CI 0.40–9.68; *p* = 0.39), whereas necrotic lesions had a significantly lower diagnostic yield (OR = 5.23, 95% CI 1.72–15.88; *p* = 0.001). Diagnostic yield was highest in cases without a circumscribed lesion (100%, 6/6). No significant difference in diagnostic yield was observed between 16 and 18G core needle biopsies (OR = 0.77, 95% CI 0.21–2.84; *p* = 0.698). Diagnostic yield increased descriptively with the number of biopsy samples obtained, reaching 98.67% (74/75) with three samples (comparison 1 vs. 3 samples: OR = 0.13, 95% CI 0.02–1.12; *p* = 0.031; Table 3). Diagnostic yield in analgosedation was significantly higher than under local anesthesia (OR = 0.25, 95% CI 0.08–0.86; *p* = 0.019) (Table 2, Fig. 3).

## Discussion

This registry-based analysis of image-guided splenic biopsies provides important insights into current practice and outcomes. We observed a consistently high technical success rate (99.00%) and diagnostic yield (90.83%), confirming the effectiveness of percutaneous spleen biopsy. The overall complication rate was low (5.00%), with major complications occurring in approximately 2.50% of cases, consistent with previous published data. McInnes et al. reported a pooled major complication rate of 2.2%, decreasing to 1.3% when excluding needles larger than 18G [10]. Similarly, Olson et al. observed an overall complication rate of 8.2%, including 1.0% major complications. [7]. Sangiorgio et al. reported no major complications and minor complications in 6.7% of cases [2]. More recently, Kavandi et al. described bleeding in approximately 8% of procedures [9].

Notably, nearly all procedures were performed in an inpatient setting (94.25%), whereas only a small fraction (5.75%) were conducted as outpatient interventions. Current practice favors inpatient or short-observation settings due to the risk of delayed hemorrhage [7, 10]. There is no robust evidence supporting routine outpatient management of image-guided splenic biopsy.

Although no dedicated national guidelines for image-guided splenic biopsy exist in Germany, available meta-analyses support the safety and diagnostic accuracy of these procedures, reporting complication rates comparable to those observed for kidney biopsies [7, 16].

In our cohort, complication rates were higher for inpatient procedures compared with outpatient biopsies; however, this finding is most likely influenced by selection bias, as outpatient biopsies were presumably performed in carefully selected patients with a lower anticipated bleeding risk.

When comparing image guidance modalities, complication rates were comparable between CT- and ultrasound-guided procedures, suggesting that modality selection is primarily driven by lesion characteristics and technical feasibility rather than safety considerations.

No significant differences in complication rates were observed in relation to pathological coagulation parameters. However, previous studies have demonstrated an association between an increased bleeding risk and pathological platelet counts [17]. International guidelines from the Society of Interventional Radiology emphasize individualized risk assessment and recommend specific thresholds for platelet count, international normalized ratio, and anticoagulant management in the setting of image-guided interventions [18, 19].

Procedures performed under local anesthesia were associated with significantly higher complication rates compared with those performed under analgesic sedation; however, this observation should be interpreted with caution, as confounding factors and selection bias cannot be excluded. Evidence from lung biopsy procedures suggests that controlled breathing and improved patient cooperation may enhance targeting accuracy and reduce complication rates.

Technical success was high across all cases. Diagnostic yield remained consistently high across settings and imaging modalities, in line with previous reports [7, 20]. The use of an 18G needle was not associated with a significantly higher diagnostic yield compared to 16G needles; however, its consistently high diagnostic performance indicates that 18G needles are sufficient for reliable histopathological diagnosis. This is consistent with previous reports demonstrating high diagnostic yield and low complication rates for 18G splenic biopsies [21, 22]. Diagnostic yield increased with the number of biopsy samples obtained, reaching its maximum with three samples.

As a registry-based study, this analysis has several limitations. Data completeness relied on site-reported documentation, and optional variables were not consistently captured across participating centers. Selection bias cannot be excluded, particularly with respect to outpatient procedures, which were likely performed in patients with a lower anticipated risk profile. In addition, the relatively small number of outpatient biopsies limited the statistical power of subgroup analyses.

No central histopathological review was performed, and complications were classified according to SIR criteria rather than the more recent CIRSE-based standards. Furthermore, relevant procedural details—such as post-biopsy observation duration, specific biopsy indications, and operator-related factors—were not available in the registry. Thresholds for defining pathological coagulation parameters were not standardized and may have varied between centers. Finally, underreporting of complications cannot be excluded, representing a potential source of reporting bias. Therefore, the observed associations should be interpreted with caution and not considered causal. Despite these limitations, the large sample size, multicenter design, and real-world nature of the registry provide valuable insights into routine clinical practice and outcomes of image-guided splenic biopsy.

## Conclusion

Image-guided percutaneous splenic biopsy is a safe and effective diagnostic procedure in routine clinical practice, demonstrating high technical success and diagnostic yield in a large multicenter registry cohort. Although splenic biopsies are widely considered a high-risk intervention, the overall and major complication rates observed in this study were low. Lower complication rates and higher diagnostic yield were observed under analgosedation compared with local anesthesia. Diagnostic yield increased with the number of biopsy samples obtained, with a maximum observed at three samples. These findings may contribute to improved diagnostic performance while maintaining procedural safety.
